# Mechanical properties of MDCK II cells exposed to gold nanorods

**DOI:** 10.3762/bjnano.6.21

**Published:** 2015-01-20

**Authors:** Anna Pietuch, Bastian Rouven Brückner, David Schneider, Marco Tarantola, Christina Rosman, Carsten Sönnichsen, Andreas Janshoff

**Affiliations:** 1 Institute of Physical Chemistry, Tammannstr. 6, University of Goettingen, 37077 Goettingen, Germany; 2 Max Planck Institute for Biology of Ageing, Joseph-Stelzmann-Strasse 9b, 50931 Cologne, Germany; 3 Max-Planck-Institute for Dynamics and Self-Organization (MPIDS), Laboratory for Fluid Dynamics, Pattern Formation and Biocomplexity, Am Fassberg 17, 37077 Goettingen, Germany; 4 Institute of Physical Chemistry, Duesbergweg 10–14, University of Mainz, 55128 Mainz, Germany

**Keywords:** atomic force microscopy, CTAB, gold nanorods, membrane tension, MDCK II cells, QCM

## Abstract

**Background:** The impact of gold nanoparticles on cell viability has been extensively studied in the past. Size, shape and surface functionalization including opsonization of gold particles ranging from a few nanometers to hundreds of nanometers are among the most crucial parameters that have been focussed on. Cytoxicity of nanomaterial has been assessed by common cytotoxicity assays targeting enzymatic activity such as LDH, MTT and ECIS. So far, however, less attention has been paid to the mechanical parameters of cells exposed to gold particles, which is an important reporter on the cellular response to external stimuli.

**Results:** Mechanical properties of confluent MDCK II cells exposed to gold nanorods as a function of surface functionalization and concentration have been explored by atomic force microscopy and quartz crystal microbalance measurements in combination with fluorescence and dark-field microscopy.

**Conclusion:** We found that cells exposed to CTAB coated gold nanorods display a concentration-dependent stiffening that cannot be explained by the presence of CTAB alone. The stiffening results presumably from endocytosis of particles removing excess membrane area from the cell’s surface. Another aspect could be the collapse of the plasma membrane on the actin cortex. Particles coated with PEG do not show a significant change in elastic properties. This observation is consistent with QCM measurements that show a considerable drop in frequency upon administration of CTAB coated rods suggesting an increase in acoustic load corresponding to a larger stiffness (storage modulus).

## Introduction

The interest in gold nanoparticles (NP) for biomedical applications in the field of nanomedicine results from both their therapeutic and diagnostic potential based on their tuneable size in the range of 1–100 nm [1–5]. Being in the size-regime of cellular components such as DNA and proteins, nanoparticles are capable to overcome native dielectric barriers like the cell membrane rendering them prime candidates for multifunctional carriers [6–8]. Potential applications encompass selective drug delivery, photothermal therapy, reporters for biosensors and the use as contrast agents [5,9]. Targets can be addressed specifically by functionalization of the particle surface (DNA, proteins, antibodies) with functional groups using self-assembly techniques relying on gold–thiol interaction. Since these NPs are engineered to interact with living cells it is essential to prove if there is no adverse impact on cell viability [5,10]. Prerequisite for successful medical applications is the design of biocompatible NPs that do not impair with cell viability, proliferation, and adhesion. Therefore assessing the cytotoxicity of nanoparticles is pivotal for nanoparticle research in general [11]. In vitro nanocytotoxicity studies are therefore necessary to minimize possible risks in the context of human exposure to nanoparticles. Hence, biosensors with high sensitivity, selectivity, fast real-time readout, and non-invasiveness are desirable design criteria for screening toxicity of nanoparticles varying in size, shape, and surface functionalization. Most cytotoxicity assays, however, such as MTS (3-(4,5-dimethylthiazol-2-yl)-5-(3-carboxymethoxyphenyl)-2-(4-sulfophenyl)-2*H*-tetrazolium) or MTT (3-(4,5-dimethylthiazol-2-yl)-2,5-diphenyltetrazolium bromide) rely on viability readouts detecting the existence of active enzymes such as NAD(P)H-dependent cellular oxidoreductase enzymes. More advanced techniques, like electric cell-substrate impedance sensing (ECIS) or quartz crystal microbalance measurements monitor the vertical cell motility, i.e., dynamic changes of the cell-substrate distance, as a reporter for cell viability [1,10–15].

Mechanical properties of cells mirror the environment such as substrate properties including topography and stiffness [16–18]. Besides, also chemical cues can produce substantial changes in membrane or cytoskeletal mechanics and dynamics providing an excellent means to assess the impact of external stimuli such as nanoparticles either attached to the plasma membrane or within the cytosol [5]. Rheological properties of epithelial cells are mainly determined by the plasma membrane associated with the underlying cell cortex. The contractile actomyosin cortex is a key feature in many dynamic cellular processes like cell migration, proliferation and tissue formation [19–20]. Mechanical behavior of living cells can be monitored spatially resolved in a concentration and time dependent manner using scanning probe techniques. It is possible to investigate local cellular elastic properties under physiological conditions using atomic force microscope (AFM) by taking force curves at each spot the probe touches the sample surface. These force indentation curves are frequently subject to regression analysis employing Hertzian contact models that permit to assess the cell’s Young’s modulus. The modulus bears invaluable information about cellular properties like the cytoskeleton or the plasma membrane [21]. Alternatively, mechanical properties of cells in response to nanoparticle exposure can be monitored time resolved by the quartz crystal microbalance with dissipation monitoring (D-QCM) [14,22–23]. The QCM-method records simultaneously the resonance frequency and dissipated energy of the quartz crystal covered with cells and reveals information about the viscoelastic properties of these cells as well as the distance from the quartz surface [24]. In the work presented here we investigated the influence of gold nanoparticles on the elasticity of the epithelial cell line MDCK II probed by AFM and QCM. The combination of these two techniques allows to monitor the influence of nanoparticles on the elastic properties of MDCK II cells both from the apical and basal side. The data permits to compare mechanics of cells exposed to either cetyl-trimethylammonium bromide (CTAB) or biocompatible polyethylene glycol (PEG) coated gold nanoparticles in different concentrations. We also examined structural rearrangement of the cytoskeleton via fluorescence microscopy and by that tried to gain a deeper understanding of how gold nanoparticles impact cell mechanics.

## Results and Discussion

**Figure 1:**
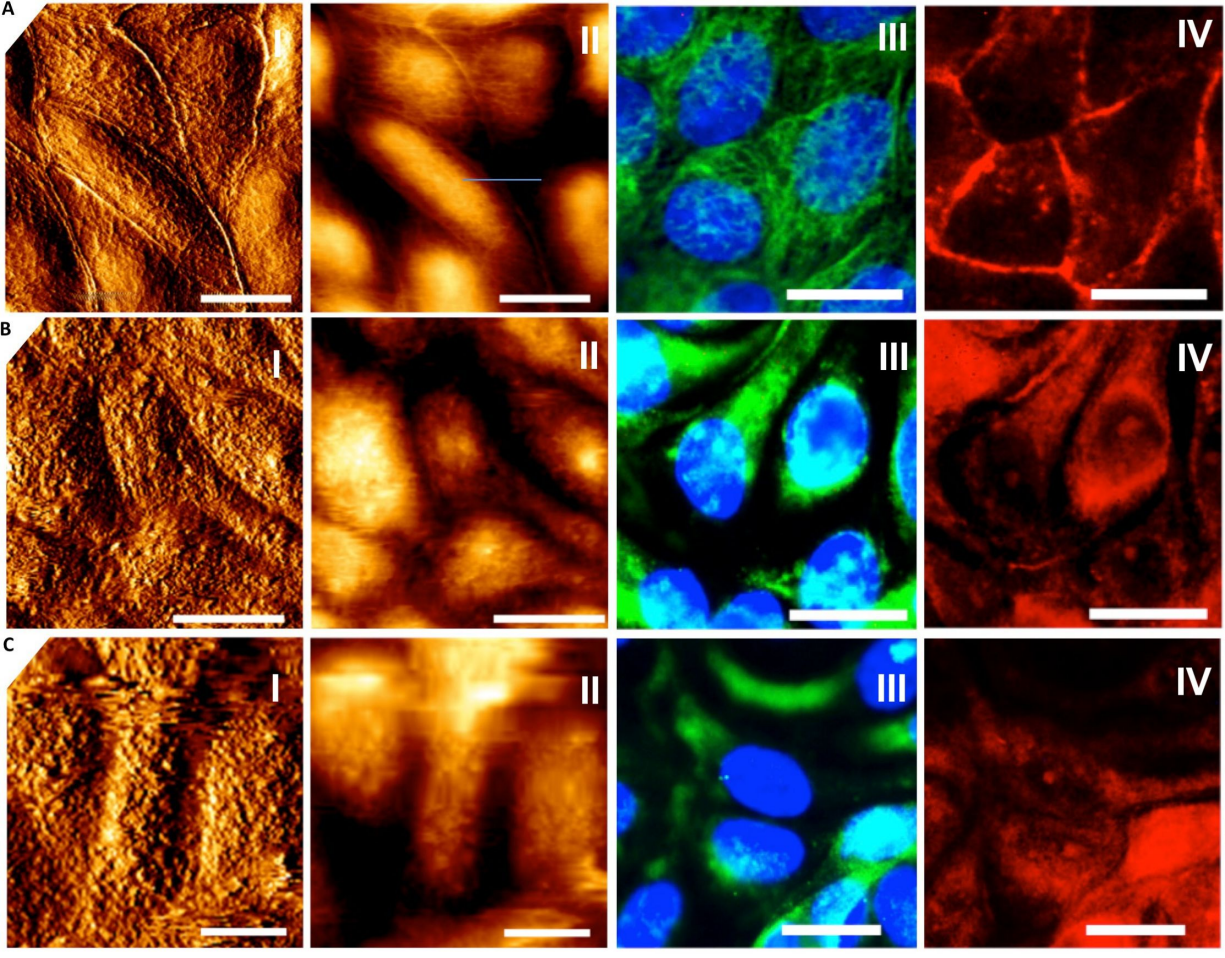
Confluent MDCK II cells treated with different concentrations of CTAB functionalized gold nanorods. A: untreated cells. B: cells incubated for 24 h with 6 μg/mL CTAB rods. C: cells incubated for 24 h with 24 μg/mL CTAB rods. I: AFM contact mode height images. II: deflection images. III: fluorescence microscopy images of microtubules (green) and nucleus (blue). IV: fluorescence microscopy images of F-actin filaments (red). Scale bar: 10 μm.

Figure 1 shows microscopy (AFM and fluorescence microscopy) images of a confluent MDCK II monolayer treated with CTAB-coated gold nanorods. CTAB is necessary to keep the particles in solution preventing precipitation due to aggregation. The AFM images (Figure 1 A–C, I+II) clearly show that the topography of the cells changes due to exposure to CTAB-rods. The surface becomes rougher and the cell-cell-borders vanish. The height of the cells decreases by approximately 1 μm (from 3–4 μm of untreated cells). Immunostaining of microtubles (Figure 1 A–C, III) and actin-filaments (Figure 1 A–C, IV) reveals that with increasing concentration and incubation time (data not shown) of CTAB-nanorods disassembly of the filaments occurs concomitant with an increase in viability loss. Cytotoxicity studies using ECIS and MTS tests show that at these concentrations the cells are no longer viable, which we largely attribute to the loss of cytoskeleton integrity and production of ROS species [10,13].

**Figure 2:**
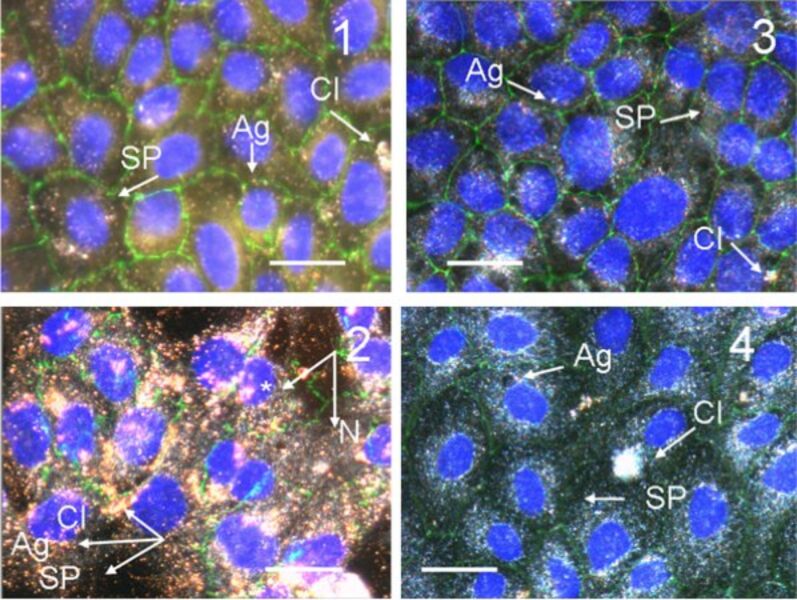
Dark-field/fluorescence microscopy image (overlay) of confluent MDCK II cells treated with gold nanorods after 24 h. Image 1: Cells exposed to 0.14 μg/mL CTAB coated gold nanorods per mL. Image 2: Cells exposed to 2 μg/mL CTAB coated gold nanorods per mL. Image 3: Cells exposed to 4.0 μg/mL COOH-PEG coated gold nanorods per mL. Image 4: Cells exposed to 4.0 μg/mL NH_2_-PEG coated gold nanorods per mL. The images are overlayed with a fluorescence images using DAPI (nucleus, blue, *) and ZO-1 (cell borders, green) staining. Arrows indicate the particles (SP: single particle; Ag: aggregate; Cl: cluster, N: nucleus). We refer to clusters as very large aggregates of nanorods. Scale bar: 20 μm.

Dark-field microscopy is an excellent tool to visualize gold nanoparticles in cells due to their light scattering ability. Figure 2 shows a confluent MDCK II monolayer after incubation with CTAB-coated gold nanorods at different concentrations. Particles as well as aggregates are easily discernible due to their plasmon resonance. The particles arrange predominantly around the nucleus but are usually not found inside the nucleus. Recently, we carried out optical dark-field microscopy together with transmission electron microscopy to quantify the uptake of gold nanoparticles into MDCK II cells as a function of shape, stabilizing agent, and surface charge [25]. We found that CTAB-coated particles are easily accumulated within cells, while PEG coatings inhibit uptake significantly. This is also reflected in the lack of cytotoxicity of PEGylated particles. Interestingly, we also found that spherical particles are more toxic than rod-like ones of the same size and with identical surface functionalization [13].

In contrast to CTAB-coated gold nanorods PEG-coated nanorods do no visibly change the cells’ cytoskeleton albeit the particles still enter the cells to some extent. The images of Figure 2 (panel 3/4) show that the particles predominantly arrange around the nucleus once they entered the cell similarly to what is observed for CTAB-coated rods. We know from our previous studies that PEG coating primarily limits their entry in the cells. We found a significantly higher number of CTAB-coated nanoparticles inside the cells (*>*2000 particles per cell) compared to NH_2_-PEG coated ones (around 200 particles per cell) [13,25].

As the cytoskeletal integrity of epithelial cells was shown to be corrupted by gold-nanoparticle exposure in these previous publications, we decided to monitor viscoelastic changes and metabolically driven shape fluctuations in real-time by means of acoustic and impedance-based sensors like QCM and ECIS; the latter furthermore enabled us to monitor the epithelial barrier function and therefore cell–cell junction dynamics. All these parameters showed a rapid decrease within the first 2.5 h and either were completely abolished after 24 h or indicated a recovery to the initial level within 48 h, as for the PEGylated particles. In terms of signaling, for both CTAB spheres and rods, we found within 24 h after treatment a reduction of mitochondrial activity (by MTS or LDH) as well as the activation of reactive oxygen species [13,25].

**Figure 3:**
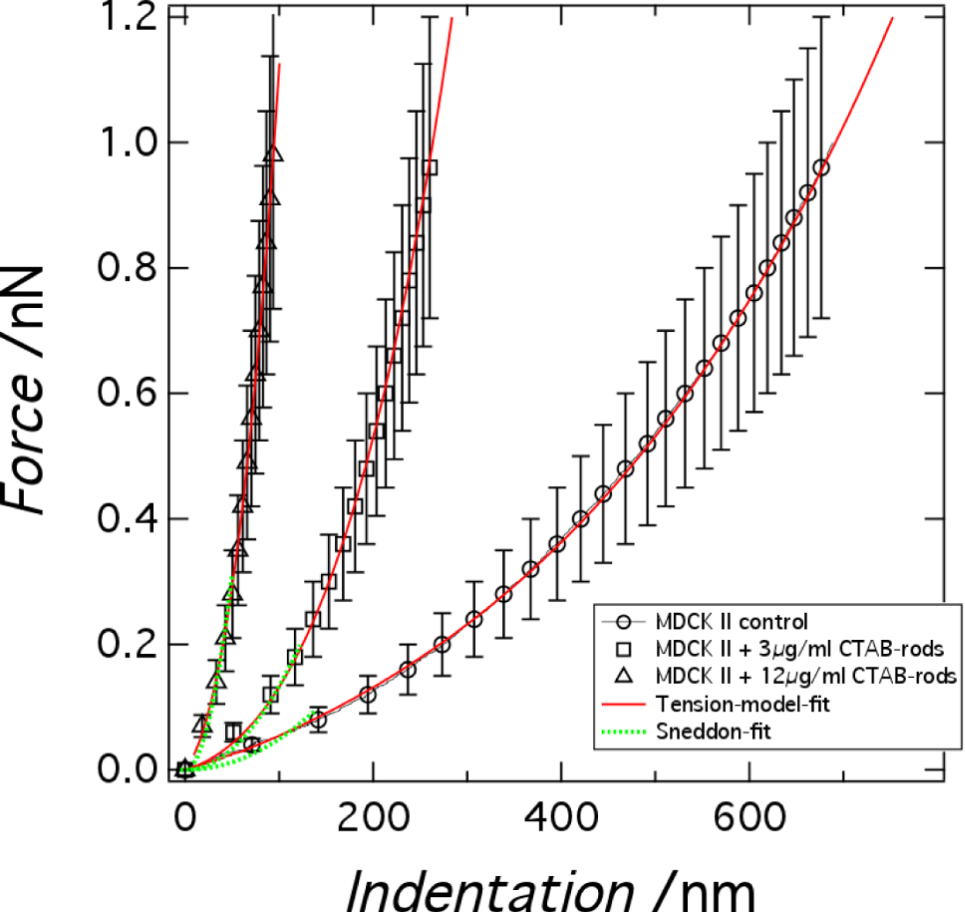
Mechanical analysis of confluent MDCK II cells. Averaged force indentation curves (*n* > 60) obtained from indentation of confluent MDCK II cells (cell center) treated with different concentrations of CTAB functionalized gold nanospheres after 24 h of incubation (see legend). The force curves are fitted to a Hertzian contact model (green) and tension model (red) as described in the text.

Cellular mechanics plays an important role in many biological processes comprising cell adhesion, migration, growth, oncogenesis and tissue formation [19,26]. For instance, it has been shown that the elastic response of cells may correlate with their metastatic potential, in which malign cells are softer than benign ones upon deformation with an external probe [18]. It is therefore conceivable that besides environmental cues also adhesion and uptake of nanoparticles is reflected in the mechanical properties of cells. Figure 3A shows averaged force indentation curves performed on the center of confluent MDCK II cells. Two different models were used to extract mechanical parameters from these data. The first one uses Hertzian contact mechanics (Sneddon model for conical indenters) providing a single parameter, the Young’s modulus of the cell (see Materials and Methods section). The range of validity is limited to only a few hundred nanometers (green dotted lines in Figure 3). Due to the well-known shortcomings of Hertzian mechanics to describe the elasticity of cells we also used a recently introduced tension model treating the cells as a liquid droplet (red continuous lines in Figure 3) [27–28].

According to the tension model we consider the cell as an isotropic elastic shell that produces a restoring force in response to indentation with a conical indenter originating from two sources, linear elasticity due to area dilatation and pre-stress (constant tension). Pre-stress is mainly generated by contractile actomyosin, strong adhesion at the cell-borders and interaction of the plasma membrane with the cytoskeleton. Bending, however, plays a minor role and can therefore be neglected. Tension *T* of the plasma membrane/cortex shell can be written as [27]:

[1]
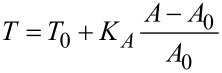


with *T*_0_ the pre-stress and *K_A_* the area compressibility modulus of the shell giving rise to a nonlinear force–indentation curve. *A* denotes the actual surface area at a given indentation depth and *A*_0_ the surface area prior to indentation.

**Figure 4:**
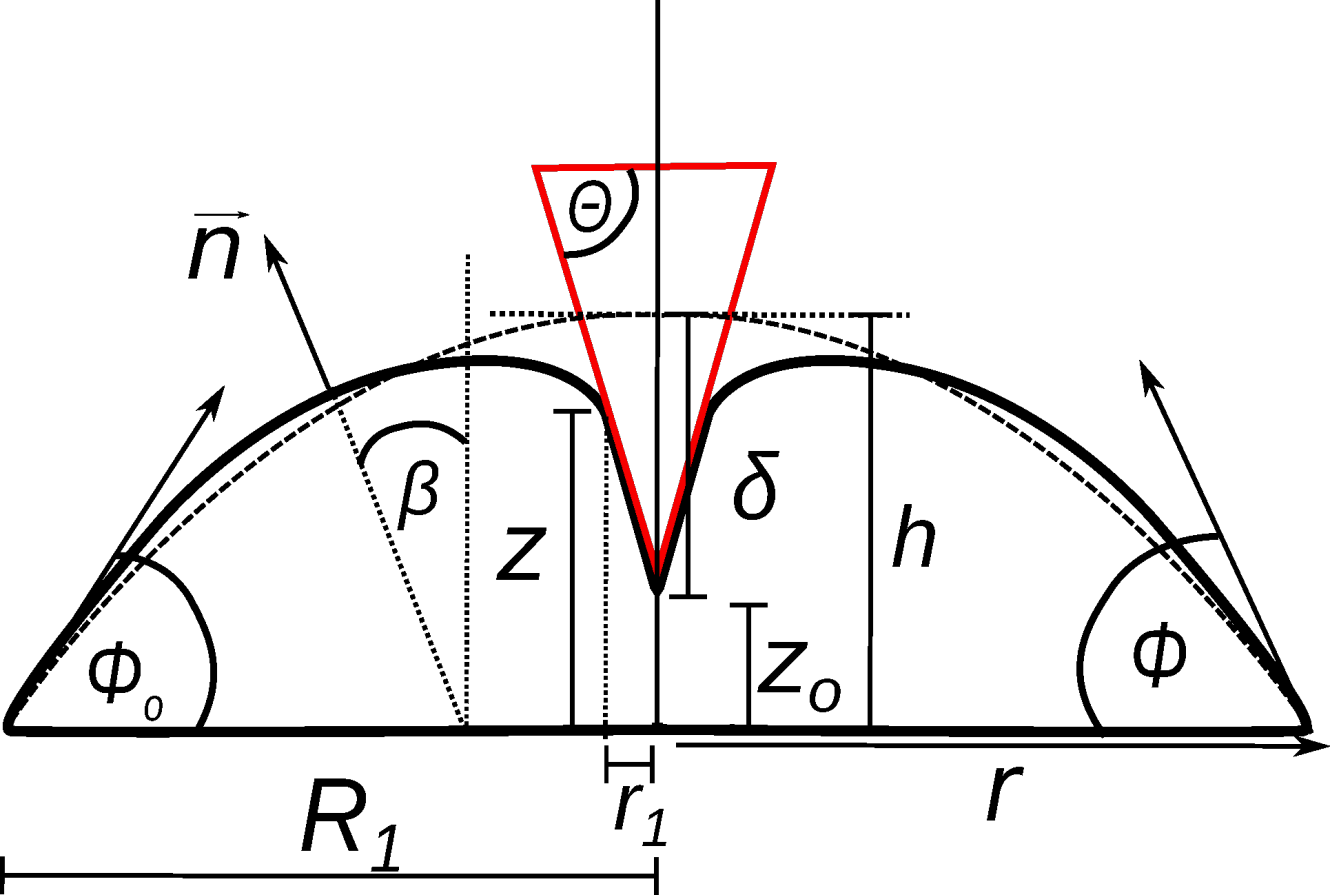
An adherent cell or apical membrane of an epithelial cell in confluent environment represented by a spherical cap (dotted line) subject to indentation using a conical indenter (continuous line). Illustration of parameters used in the tension model [28].

Static equilibrium can be expressed by the Young–Laplace equation, which describes the pressure difference across the fluid interface as a function of surface tension *T* and mean curvature. The task is to determine the actual 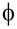 and *r*_1_ (Figure 4), which constitute essentially the shape of the indented cell assuming that the volume of the cell is preserved during the indentation process. Indentation inevitably leads to an area increase that produces additional tension. The fit to the force-indentation curves provides two parameters, the pre-stress *T*_0_ and the area compressibility modulus *K_A_*. For pure lipid bilayers a *K_A_* value of 0.1–1 N/m is usually found due to the lateral inextensibility of the plasma membrane depending on the cholesterol content. If the value is smaller than 0.1 N/m we assume the presence of excess membrane area. If the value is significantly larger we assume that not all of the geometrical area of the spherical cap can be recruited to resist indentation. Therefore, *K_A_* becomes an apparent area compressibility modulus reflecting the recruitable surface area of the plasma membrane.

**Figure 5:**
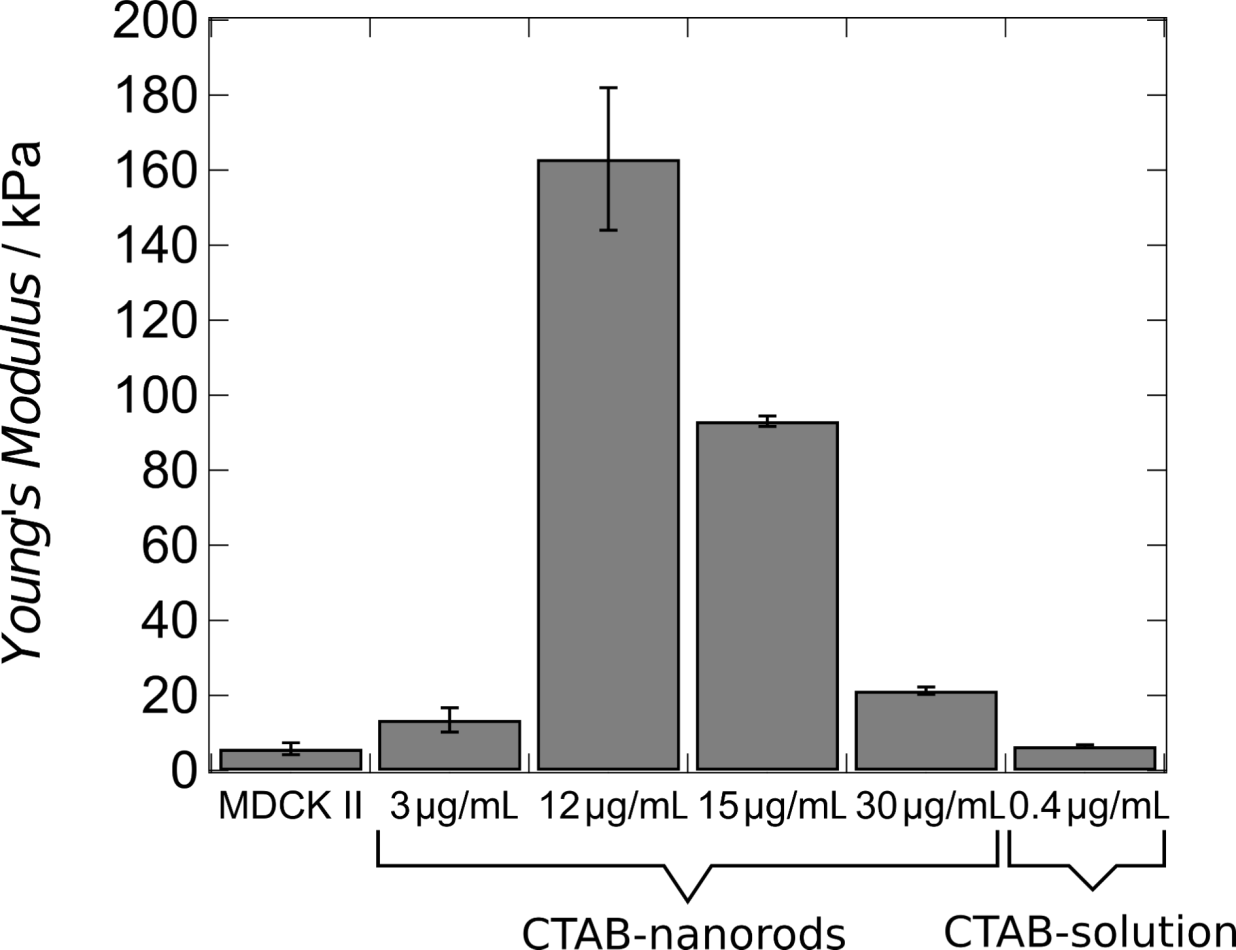
Young’s moduli of MDCK II cells treated with different concentrations of gold nanorods and CTAB solution.

The tension model allows to describe the elastic response to indentation also at large strain capturing the nonlinear stress response by adding a stretching term. We assume that the cell–cell contacts connected to the contractile F-actin ring, which are also visible in the AFM images (Figure 1), serve as tension generating boundaries as opposed to a single cell, where the boundary is given by the substrate itself. We justify this approach also by AFM topography images of confluent untreated MDCK II cells that reveal a distance of the apex of the cell to the cell–cell boundaries, i.e., the height of the apical cap, of approximately 1 micrometer. While for untreated cells we found a pre stress of *T*_0_ = 0.7 ± 0.1 mN/m and an area compressibility modulus of *K_A_* = 0.8 ± 0.02 N/m assuming a radius of the cap of *R*_1_ = 12 μm and a contact angle of 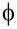
_0_ = 20° (Figure 4) , cells exposed to CTAB coated gold nanorods even at low concentration of 3 μg/mL display a considerable increase in both pre-stress (*T*_0_ = 1.2 ± 0.1 mN/m) and area compressibility modulus (*K_A_* = 14.2 ± 0.1 N/m). Finally, at 12 μg/mL CTAB nanorods we observe a maximal pre-stress of *T*_0_ = 3.0 ± 0.1 mN/m and an area compressibility modulus of *K_A_* = 250 ± 1 N/m. Considering these extremely stiff cells at high CTAB coated gold nanorods concentrations as liquid droplets is probably no longer justified. It is difficult to explain these extraordinary high values in terms of cortical or even membrane tension and inextensibility of the plasma membrane alone. Although it is conceivable that excessive endocytosis leads to a loss of excess surface area the collapse of the plasma membrane on the elastic and considerably stiff actomyosin cortex is also important to explain the mechanical response. Therefore, we will base the following discussion mainly on the results of employing conventional contact models based on Hertzian mechanics expressing the mechanical properties as a single parameter, the Young’s modulus. However, the fits of the liquid droplet model describe the data very well and show the same trend as the more conventional contact models assuming a semiinfinite elastic continuum. Essentially, all models point unequivocally towards stiffer cells in response to addition of CTAB coated particles. This means that regardless of the mathematical description, the cells become stiffer if exposed to CTAB-coated nanoparticles with increasing concentration up to 12 μg/mL. Concentrations larger than 12 μg/mL lead to softening of the cells most likely due to loss of vitality and disintegration of the cytoskeleton. Compared to fixation with glutardialdehyde, leading to a Young’s modulus of *E* = 25 kPa [22], the cells are even stiffer after addition of 12 μg/mL CTAB coated gold nanoparticles (*>*100 kPa). Importantly, this effect seems to be independent of particle shape. Using CTAB-coated gold nanospheres with a mean diameter of 43 nm we found that at a particle concentration of 3 μg/mL already results in a Young’s modulus of *E* = 42 kPa (Figure 5). Higher particle concentrations are already toxic and the cells start disintegrating, while smaller concentrations (0.5 μg/mL) show a reduced cell stiffness (*E* = 1 kPa).

In contrast, if PEGylated gold nanrods are added to the confluent cell monolayer, the mechanical response of the cells is negligible compared to the control. For instance, using NH_2_-PEG coated particles (23 μg/mL) the Young’s modulus shifts from *E* = 5.7 ± 0.1 kPa for untreated cells to *E* = 4.3 ± 0.3 kPa. A coating with COOH-PEG possessing the opposite charge results in a mean Young’s modulus of *E* = 3.9 ± 0.1 kPa. However, it is important to note that uptake of PEGylated nanoparticles is strongly reduced compared with CTAB-coated particles. Therefore, we cannot compare the impact of particles with different surface functionalization on cellular mechanics directly.

From the results so far it seems to be conceivable that CTAB alone is responsible for the apparent stiffening of the cells. Therefore, we added CTAB (in the absence of gold nanoparticles) to confluent MDCKII cells. We found indeed that the cells stiffen but not by the same extent as in the presence of CTAB-coated particles. Using a CTAB (0.4 μg/mL) concentration that represents the number of CTAB molecules which can, in principle, be released from the particle surface assuming a concentration of 12 μg/mL, we only found a Young’s modulus of *E* = 6.5 ± 1 kPa. Fluorescence microscopy images (staining of actin and microtubules) as well as AFM images (topography) reveal that the cells remain largely intact after addition of pure CTAB solution. Therefore, the impact of CTAB-coated gold nanoparticles on cellular mechanics is a combined effect of CTAB molecules displayed by particles inside the cell. Since the particles are essentially positively charged it is conceivable that they are wrapped by the plasma membrane and thereby consuming all excess surface area. A reduction of surface area immediately leads to apparent cell stiffening at larger strains. Enforced endocytosis leads to a decrease in overall membrane area that causes increased resistance against area dilatation. Pre-stress is also found to be increased after administration of CTAB-coated particles, which hints twoards actin remodelling and higher contractility of the actomyosin cortex.

The substantial increase in stiffness of MDCK II cells is especially surprising since immunostaining of the cytoskeleton reveals that the cells’ cytoskeleton disintegrates in good accordance with our previous cytotoxicity studies. F-actin filaments are severed and the microtubules network is destroyed. However, comparing the situation to addition of small molecular inhibitors such as cytochalasin D that visibly produce a similar depolymerization of F-actin, the cells are considerably (10×) softer (*E* = 0.4 ± 0.2 kPa) compared to the untreated control.

**Figure 6:**
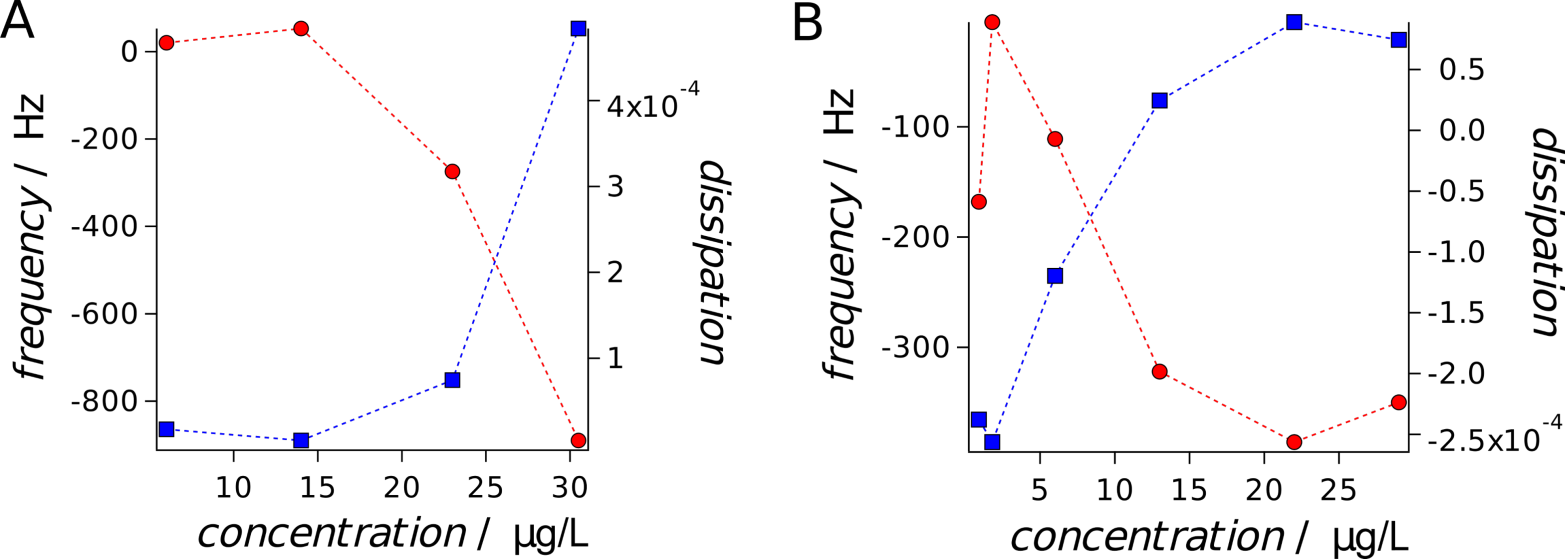
Equilibrium frequency and dissipation change (after 24 h of incubation) of a confluent MDCK II cell monolayer cultured on a 5 MHz quartz (fundamental frequency) as a function of CTAB-coated gold nanorod (A) and CTAB-cated gold nanosphere (B) concentration.

One possible explanation of the findings is that the plasma membrane and cortex are partly dissolved and we are instead probing the nucleus which is a much stiffer organelle. Another hypothesis could be that the positively charged CTAB coated gold nanorods lead to removal of excess plasma membrane and generate a reinforced shell. The latter is supported by the fact that the height of the cells is reduced after addition of CTAB-coated particles by about 1 μm indicative of a collapsed structure. Further arguments in favor of this idea and against the former interpretation that we only probe the nucleus or the substrate are our D-QCM measurements, which also show a tremendous stiffening of the cells comparable to what is found if the cells are fixed with glutardialadehyde (GDA). Fixation of cells with GDA results in a shift to lower resonance frequency by a few hundred Hertz, while dissipation grows [14]. The same is observed for CTAB-coated gold nanorods and nanospheres (Figure 6). In good accordance, elasticity measurements of MDCK II cells after GDA fixation with an AFM provides a Young’s modulus of at least 25 kPa [22]. It requires, however, larger particle concentration to provoke a change in acoustic load of the cells cultured on the resonator. We observe almost no effect up to 6 μg/mL of CTAB coated nanorods, while the maximal response is found for 30.5 μg/mL. This is probably due to the fact that the QCM detects changes in the viscoelasticity close to the resonator’s surface, which affects the basolateral side of the cell monolayer. Hence, it probably requires larger numbers of particles to generate a change in cell elasticity. In contrast, AFM indentation experiments target mainly the apical part of the cell monolayer. The values for the Young’s modulus obtained from QCM measurements and those measured by cell indentation are not directly comparable since the frequency by which the QCM acquires data (5 MHz) is 5–6 orders of magnitude larger than that of acquiring force curves. The cells therefore appear substantially stiffer on the resonators surface as compared to the AFM experiments. Moreover, the drop in frequency also depends on the distance between cell and quartz crystal. A smaller distance results in a larger frequency decrease. Interestingly, we found that both mass load (change in resonant frequency) and dissipation (representing energy loss) increase upon administration of CTAB coated gold nanorods. The response time of the cells to administration of particles is fairly fast (few hours) and depends heavily on particle concentration. We carried out concentration-dependent QCM measurements and found that damping (dissipation) increases steadily from 2.5 to 25 μg/mL until eventually leveling off (Figure 6).

Generally, dark-field micrographs showed that particles are homogeneously distributed within the cell interior with a trend to accumulate around the periphery of the nucleus. However, we never observed particles inside the nucleus. On some TEM images, particles are located in close proximity to the inner cell membrane [13]. In essence, we observed uptake and aggregation of CTAB coated particles almost immediately after addition, at least within a few hours. Only few gold nanorods or spherical particles were found inside MDCK II cells if they were functionalized with PEG.

## Conclusion

In conclusion, we found that adding CTAB coated gold nanorods and spherical particles to confluent MDCK II cells results in a substantial stiffening of the cell that we attribute to a loss of plasma membrane due to enforced endocytostis. Frequently we found macropinocytosis and aggregation of CTAB-particles inside the cells. Membrane permeabilization was not observed. We propose that the positively charged particles initially adsorb on the negatively charged plasma membrane and therefore enter the cell triggered by the large adhesion energy. Sometimes also larger aggregates are found inside the cells. Endocytosis eventually leads to reduction of the plasma membrane area. This area, however, is needed to resist large strains, i.e., area dilatation that occur upon indentation. Higher concentrations of particles eventually lead to disassembly of the actin cytoskeleton and therefore to softening of the cells. PEGylated particles do not provoke a change in cell elasticity but this is simply because they do not enter cells as efficiently.

## Experimental

MDCK II cells were maintained in Earle’s minimum essential medium supplemented with 4 mM glutamine, 0.2 mg/mL of both penicillin and streptomycin (Biochrom, Berlin, Germany), 10% (v/v) fetal calf serum (PAA, Pasching, Austria) in a 5% CO_2_ humidified incubator (HERA cell 150, Heraeus, Germany). Cells were subcultured weekly after reaching confluence by washing with PBS, followed by trypsinization and centrifugation at 110*g*.

### Particle synthesis and characterization

Gold nanorods and nanospheres were prepared as described previously following the seeded growth method [25]. First, seeds were prepared by adding 0.6 mL of ice-cold 0.010 M sodium borohydride (NaBH_4_) to 10 mL solution of 0.1 M cetyltrimethylammonium bromide (CTAB) equipped with 50 μL 0.1 M tetrachloroauric acid (HAuCl_4_) under vigorous stirring. Second, rods were formed by adding 12 μL of seed solution to a growth solution consisting of 75 μL 0.1 M HAuCl_4_, 10 mL of 0.1 M CTAB, 7 μL of 0.04 M silver nitrate (AgNO_3_), and 105 μL of 0.08 M ascorbic acid. Nanoparticle size was controlled by transmission electron microscopy (TEM). We determined a length of 38 ± 6.5 nm and a width of 17 ± 3 nm for nanorod and a diameter of 43 nm for spheres [25]. Concentrations of gold nanorods were obtained from UV–vis spectroscopy using their optical extinction value at 400 nm and a molar extinction coefficient of 1.1 × 10^9^ Lmol^−1^cm^−1^ assuming the aforementioned particle size found by TEM. In order to replace the CTAB, functionalized polyethylene glycol thiols (X-PEG-SH; X = COOH, NH_2_, CH_3_O, MW: 5,000 g/mol) were self-assembled on the gold surface. Nanoparticle pellets were incubated overnight with 100 μL of an aqueous 2 mM solution containing 75% NH_2_-PEG-SH and 25% CH_3_O-PEG-SH (NH_2_-PEG-particles) or 75% COOH-PEG-SH and 25% CH_3_O-PEG-SH (COOH-PEG-particles), respectively. The following day, excess PEG in solution was removed by centrifugation of the suspension and PEGylation was confirmed by gel electrophoresis [10,13,25]. Concentration of particles is given as concentration of gold in μg/mL or particle number per mL.

### Fluorescence and dark field imaging

Immunostaining for fluorescence microscopy was used to study the alteration of the cytoskeleton upon nanoparticle exposure. Therefore, MDCK II cells were fixed after every AFM experiment by immersing the cells into a (−20 °C) acetone/methanol mixture (1:1) for 10 min. Afterwards, the cells are rinsed three times with PBS. Incubation with staining solution was carried out following the manufacture’s recommendation. For F-actin staining Alexa Fluor 546 Phalloidin (Invitrogen, Darmstadt, Germany) and for microtubules labeling Alexa 488 conjugated mouse anti-β-tubulin (BD Biosciences, Heidelberg, Germany) was used. Nucleus staining was carried out with DAPI (4’,6-diamidino-2-phenylindol, 50 ng/mL in PBS) (Sigma-Aldrich, Steinheim, Germany). Dark-field microscopy was carried out with an upright microscope with dark-field condensor (Olympus BX51) equipped with a 40× water immersion objective.

### AFM imaging and force distance curves

For AFM measurements cells were seeded onto conventional glass slides. After reaching confluency, cells are exposed to different gold nanoparticle concentrations and surface modifications for 24 h. AFM imaging was performed on a Nanowizard II AFM (JPK Instruments AG, Berlin, Germany) mounted on an inverted optical microscope (Axiovert 200, Zeiss, Oberkochen, Germany) to localize cell position. Silicon nitride cantilever (MLCT-AUHW, Bruker, Germany) were used with a nominal force constant of ≈0.05 N/m. Imaging of cells has been carried out using a fluid-heating chamber (Biocell, JPK Instrument AG, Berlin, Germany) with HEPES buffered culture medium kept at 37 °C. AFM images were performed in contact mode. Before force spectroscopy measurements the exact spring constant of the used cantilever was determined by thermal noise analysis using software provided by the manufacturer.

Local mechanical measurements were carried out on confluent MDCK II monolayers directly after imaging using the same cantilever. Force curves were collected with a *z*-scan velocity of 1 μm/s. Analysis of the elasticity modulus was done with a tool developed in our laboratory using IGOR Pro (WaveMetrics, Lake Oswego, OR, USA). To calculate *E* (Young’s Modulus) from force curves we used Sneddon’s modification of the Hertzian model for a conical tip [21,27–28]:

[2]
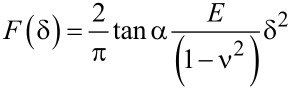


Here, *E* is the Young’s modulus, ν is the Poisson ratio of the sample (0.5), α is the half-opening angle of the AFM tip (35°) and δ the indentation depth.

An alternative approach to describe the elastic response of confluent epithelial cells to indentation with an AFM-tip is the so-called tension model that treats the cell as a liquid droplet as described above [27–28]. Adhesion of cells to the surface or to each other in a confluent monolayer leads to shapes that can best be described as capped spheres with initial contact (wetting) angles around 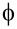
_0_ = 60°.

### Quartz Crystal Microbalance

Quartz crystal microbalance in dissipation mode (D-QCM) measurements were carried out as described before [14]. In brief, changes in resonance frequency and dissipation were recorded with a home-made D-QCM setup using 5 MHz AT-cut quartz crystals (KVG, Neckarbischofsheim, Germany) with circular gold electrodes. Cells were seeded to confluency (1× 10^6^ cells per cm^2^) directly onto the crystal mounted in a thermostatted fluid cell made from Teflon [15].
